# Plant miRNAs Reduce Cancer Cell Proliferation by Targeting MALAT1 and NEAT1: A Beneficial Cross-Kingdom Interaction

**DOI:** 10.3389/fgene.2020.552490

**Published:** 2020-09-18

**Authors:** Flaviana Marzano, Mariano Francesco Caratozzolo, Arianna Consiglio, Flavio Licciulli, Sabino Liuni, Elisabetta Sbisà, Domenica D’Elia, Apollonia Tullo, Domenico Catalano

**Affiliations:** ^1^Department of Biomedical Sciences, Institute of Biomembranes, Bioenergetics and Molecular Biotechnologies, Bari, Italy; ^2^Department of Biomedical Sciences, Institute for Biomedical Technologies, Bari, Italy

**Keywords:** cancer, MALAT1, NEAT1, nutrition, plant miRNA, long non-coding

## Abstract

MicroRNAs (miRNAs) are ubiquitous regulators of gene expression, evolutionarily conserved in plants and mammals. In recent years, although a growing number of papers debate the role of plant miRNAs on human gene expression, the molecular mechanisms through which this effect is achieved are still not completely elucidated. Some evidence suggest that this interaction might be sequence specific, and in this work, we investigated this possibility by transcriptomic and bioinformatics approaches. Plant and human miRNA sequences from primary databases were collected and compared for their similarities (global or local alignments). Out of 2,588 human miRNAs, 1,606 showed a perfect match of their seed sequence with the 5′ end of 3,172 plant miRNAs. Further selections were applied based on the role of the human target genes or of the miRNA in cell cycle regulation (as an oncogene, tumor suppressor, or a biomarker for prognosis, or diagnosis in cancer). Based on these criteria, 20 human miRNAs were selected as potential functional analogous of 7 plant miRNAs, which were in turn transfected in different cell lines to evaluate their effect on cell proliferation. A significant decrease was observed in colorectal carcinoma HCT116 cell line. RNA-Seq demonstrated that 446 genes were differentially expressed 72 h after transfection. Noteworthy, we demonstrated that the plant mtr-miR-5754 and gma-miR4995 directly target the tumor-associated long non-coding RNA metastasis-associated lung adenocarcinoma transcript 1 (MALAT1) and nuclear paraspeckle assembly transcript 1 (NEAT1) in a sequence-specific manner. In conclusion, according to other recent discoveries, our study strengthens and expands the hypothesis that plant miRNAs can have a regulatory effect in mammals by targeting both protein-coding and non-coding RNA, thus suggesting new biotechnological applications.

## Introduction

Numerous epidemiological and *in vitro/in vivo* studies indicated that plant food intake compounds or phytochemicals could directly influence various molecular pathways, reducing the risk of chronic diseases (e.g., diabetes, hypertension, cardiovascular disease, and cancer) (Issa et al., 2006; Dell’Agli et al., 2013; Upadhyay and Dixit, 2015).

Recent studies have demonstrated that some plant-/food-derived microRNAs (miRNAs), primarily acquired through food intake, accumulate in sera, tissues, and feces (Zhang L. et al., 2012; García-Segura et al., 2013; Link et al., 2019).

Mature miRNAs are a category of ubiquitous small non-coding RNAs, 16–24 nucleotides long, and evolutionarily conserved (Zhang et al., 2006). These small molecules play significant roles in the modulation of several physiopathological cellular processes such as organism development, cancer, and cancer progression. Functionally, in plants and animals, mature miRNAs linked to AGO proteins target complementary messenger RNA (mRNA) sequences leading to downregulation or completely inhibition of their expression (Bartel, 2004; Lopez-Gomollon et al., 2012).

In animals, the main element for AGO protein binding to its mRNA target is a sequence of six to eight nucleotides (seed region), located at the 5′ end of the miRNA, sometimes interrupted by a specific G-bulge. The most efficient type of seed is in positions 2–8. Two other seed types are in positions 2–7 (6mer sequence) and 3–8 (offset-6mer), but they are associated with a weaker conservation and much lower efficacy (Brennecke et al., 2005; Krek et al., 2005; Lewis et al., 2005; Friedman et al., 2009). The additional pairing of an miRNA sequence (3′ supplementary sites) in positions 12–16 can occasionally strengthen seed binding (Ameres and Zamore, 2013). Transcriptomic analysis and translation efficiency experiments indicated that miRNA-induced mRNA degradation is the leading mechanisms through which miRNAs inhibit target transcripts translation in mammals. Nevertheless, an accurate study demonstrated that miRNAs at first suppress mRNA translation and later induce mRNA degradation (Zhang K. et al., 2018).

In plants, various classes of small RNAs (sRNAs) have been reported, such as miRNAs, transactivating RNAs (ta-RNAs), and heterochromatin-associated RNA (hc-siRNA). ta-siRNAs and miRNAs are the principal categories of plant sRNAs. They regulate plant development and growth by negatively controlling the expression of their target genes, mainly through transcriptional cleavage (Singh et al., 2018). Differently from animals, plant miRNAs targeting mechanism were initially believed to be the perfect or near-perfect match of the entire miRNA to the mRNA sequences. Moreover, also the effect was believed to be different, inducing primarily translational repression of the transcripts. However, recent studies demonstrated that even if high complementarity between plant miRNA and their target mRNAs is needed for silencing effectiveness, variations from this rule are possible (Li et al., 2018a).

Concerning the target sequence positions in animal mRNAs, miRNAs were at first believed to have their targets exclusively in the 3′-untranslated region (UTR) of transcripts, whereas in plant miRNAs are in the coding sequences (CDS). Recent papers demonstrated that both plant and animal miRNAs can complement 3′UTR, 5′UTR, and CDS transcript regions, suggesting more similarities of miRNA action in both kingdoms than previously believed (Lytle et al., 2007; Schnall-Levin et al., 2010; Zhang K. et al., 2018). Size, structure, and function are very similar in plants and animals and have been preserved throughout evolution. Altogether these features might allow a cross-kingdom functional interaction. This hypothesis is supported by a growing number of papers published in the last decade (Minutolo et al., 2018; Li et al., 2018a, 2019; Link et al., 2019).

In this paper, we report the results of a study aiming to investigate if plant miRNAs with a 5′ end (positions 2–8, 2–7, and 3–8) perfectly matching the seed region of human miRNAs might target human genes with a mechanism similar to that of endogenous miRNAs. In particular, we investigated the effect of selected plant miRNAs on human genes that regulate cell proliferation and cancer.

For the first time, our results demonstrate that mtr-miR-5754 and gma-miR-4995 plant miRNAs bind the long non-coding RNAs (lncRNAs), metastasis-associated lung adenocarcinoma transcript 1 (MALAT1), and nuclear paraspeckle assembly transcript 1 (NEAT1) in a sequence-specific manner, thus reducing the stability of these oncogenic target transcripts, whose products promote cell proliferation.

## Materials and Methods

### Computational Analysis

#### Data Sources and Computational Resources

For the computational analysis, we developed a local data warehouse (implemented in MySQL DBMS) that was used to store and integrate data extracted from public databases and data collections and to manage the data generated by our analysis.

Public sources used were miRBase (release 21; Griffiths-Jones et al., 2006), miRTarBase (release 7.0; Hsu et al., 2011), Ensembl/Gencode (release 28, GRCh38; Harrow et al., 2012), the NCBI Taxonomy (Sayers et al., 2010), and data extracted from the work of Cummins et al. (2006) on colorectal microRNAome.

Data from external databases were downloaded and integrated in the data warehouse by using extraction–transformation–loading (ETL) procedures implemented with Bash and Perl scripts. Plant data were enriched by manual annotation with taxonomic passport data of edible and Officinalis plant species available in specialized databases, i.e., EURISCO (Weise et al., 2017), Mediterranean Germplasm database^1^, and the Natural Resource Conservation Service of the United States Department of Agriculture.^2^

#### Human–Plant miRNAs Seed Comparison and Selection

**FIGURE 1:**
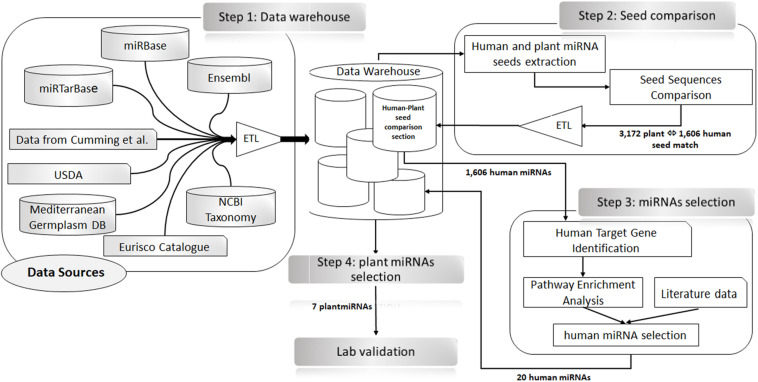
Schematic representation of the bioinformatics analysis approach for the selection of plant microRNAs (miRNAs) to be used for transfection experiments. Step 1: Data warehouse—data from external resources (as shown in the scheme) were extracted and integrated into the data warehouse using extraction, transformation, and loading (ETL) procedures. Step 2: Seeds comparison—SQL procedures were used (Issa et al., 2006) to extract, the seed regions of the human and plants miRNA considering the positions 2–7.2–8.3–8.2) to identify the perfect matches between plant and human seed sequences. The results of this analysis (i.e., the official name of the human–plant miRNA pairs, the shared seed sequences, their positions, and the whole sequence) were collected in the Human–Plant Seed Comparison section of the data warehouse. Step 3: miRNAs selection—the 1,606 human miRNAs identified in Step 2 were used to search and extract from miRTarBase the list of their target genes. Only strong experimental evidence (i.e., luciferase reporter assay, Western blotting, and RT-qPCR) were selected. The list of genes obtained was used for an enrichment analysis in Database for Annotation, Visualization and Integrated Discovery (DAVID). The results of this analysis, along with information extracted from the literature, allowed us to identify 20 human miRNAs as best candidates. Step 4: Plant miRNAs selection—the 20 miRNAs, selected in Step 3 were used to search in our data warehouse for plant miRNAs with an identical sequence at the 5′ end. In total, seven plant miRNAs were found that were used for the transfection experiments (Step 4).

To identify plant miRNAs perfectly matching human miRNA seeds (2–7, 2–8, and 3–8) in the same positions (e.g., hypothetical functional analogous), we queried our local data warehouse. The results were uploaded in a dedicated section of the local data warehouse (i.e., Human–Plant Seed Comparison section). The list of putative human miRNAs analogous to plant miRNAs was used to extract from miRTarBase the target genes validated with strong experimental evidence [i.e., luciferase reporter assay, Western blotting, reverse transcription quantitative PCR (RT-qPCR)] (Supplementary Table 1). The list of human target genes extracted was analyzed to search for gene functional enrichment in colon cancer and cell proliferation biological processes and pathways with Database for Annotation, Visualization and Integrated Discovery (DAVID) v6.8 (Huang et al., 2009a, b). To select, from the results of this analysis, the hypothetical functional analogous plant–human pairs, we considered the role of the target gene and of the targeting miRNA in a key step of the cell cycle regulation (oncogene, tumor suppressor, or biomarker for prognosis or diagnosis of colon cancer; CBD biomarkers database) (Zhang X. et al., 2018). The literature was also consulted (Piepoli et al., 2012) to substantiate our selection hypothesis. See Figure 1 for a detailed schema of the bioinformatics process.

#### Plant miRNAs Target Prediction in MALAT1 and NEAT1

The prediction of plant miRNA targets on MALAT1 and NEAT1 was carried out using miRanda (microRNA Target Detection Software) (Betel et al., 2010). As query sequences, we used the selected plant miRNAs in Table 1 and as target sequences, the transcripts of MALAT1 and NEAT1 extracted from the Vega repository of the Ensembl database (Release 68) (Hubbard et al., 2002).

**TABLE 1:** Human–plant microRNAs (miRNAs) seed comparison results.

Crop plant miRNA	Human miRNA	Hsa seed sequence	Seed match type on the 5′ region of plant miRNAs
gma-miR160	hsa-miR-1254	GCCUGG	type_2_7
gma-miR160	hsa-miR-661	GCCUGG	type_2_7
gma-miR4351	hsa-miR-450a-l-3p	UUGGGAA	type_2_8
gma-miR4368	hsa-miR-483-5p	AGACGG	type_2_7
gma-miR4995	hsa-miR-34a-5p	GGCAGUG	type_2_8
gma-miR4995	lisa-miR-34c-5p	GGCAGUG	type_2_8
gma-miR4995	hsa-miR-449a	GGCAGUG	type_2_8
gma-miR4995	hsa-miR-449b-5p	GGCAGUG	type_2_8
gma-miR5677	hsa-miR-133a-3p	UGGUCCUUGGUC	type _2_7, typ e_3_8
gma-miR5677	hsa-miR-133b	UGGUCCUUGGUC	type _2_7, typ e_3_8
mtr-miR5754	hsa-miR-25-3p	AUUGCAC	type_2_8
mtr-miR5754	hsa-miR-32-5p	AUUGCAC	type_2_8
mtr-miR5754	hsa-miR-363-3p	AUUGCAC	type_2_8
mtr-miR5754	hsa-miR-367-3p	AUUGCAC	type_2_8
mtr-miR5754	hsa-miR-92a-3p	AUUGCAC	type_2_8
mtr-miR5754	hsa-miR-92b-3p	AUUGCAC	type_2_8
zma-miR172	hsa-miR-29a-3p	AGCACCA	type_2_8
zma-miR172	hsa-miR-29b-3p	AGCACCA	type_2_8
zma-miR172	hsa-miR-29c-3p	AGCACCA	type_2_8
zma-miR172	lisa-miR-593-5p	GGCACCA	type_2_8

### Experimental Analysis

#### Cell Lines

Human neuroblastoma LAN-1-p53^–/–^, glioblastoma T98G with mutant p53, liver cancer HepG2 with p53^+/+^, colorectal carcinoma HCT116 with p53^+/+^, and HCT116 cells-p53^–/–^ were cultured in Dulbecco’s modified Eagle’s medium (DMEM) plus 10% fetal bovine serum (FBS), L-glutamine (2 mM), penicillin (100 U/ml), and streptomycin (100 μg/ml) at 37°C, 5% CO_2_.

#### Transfections of Human Cell Lines

LAN-1, T98G, HepG2, HCT116 p53^+/+^, and HCT116 p53^–/–^ cells were transfected at the confluency of 70% with the selected exogenous plant miRNAs (gma-miR-160, gma-miR-4995, gma-miR-4368, gma-miR-5677, gma-miR-4351, zma-miR-172, and mtr-miR-5754), control miRNA, which is a miRNA that has no homology to any known microRNA or mRNA sequences (Ambion; 15 μM f.c.), anti-MALAT1 and anti-NEAT1 siRNA (100 μM f.c.), or scramble siRNA using polyethylenimine (PEI, Sigma Aldrich). At the time of the experiments, cells were collected for Cell Counting Kit-8 (CCK-8) assay and for RNA extraction.

#### Cell Proliferation Assay by Cell Counting Kit-8

LAN-1, T98G, HepG2, HCT116 p53^+/+^, and HCT116 p53^–/–^ cells (5 × 10^3^) were plated in 96-well plates. 24, 48, and 72 h after transfections, quantification of cytoproliferation was measured using CCK-8 assays. The kit is based on the addition of 10 μl of highly water-soluble tetrazolium salt WST-8 [2-(2-methoxy-4-nitrophenyl)-3-(4-nitrophenyl)-5-(2,4-disulfophenyl)-2Htetrazolium,monosodium salt] to the cells for 3 h at 37°C. WST-8 is reduced by dehydrogenases in cells to give a yellow-colored product (formazan), which is soluble in the tissue culture medium. The amount of the formazan dye generated by the activity of dehydrogenases in cells is directly proportional to the number of living cells, and the absorbance is measured at 450 nm.

#### Cell Cycle Analysis

The HCT116 p53^–/–^ cell transfected with plant miRNA mix (15 μM f.c. for each miRNA) or with the control miRNA were analyzed using Tali^®^ Cell Cycle Kit (Life Technologies). The cells were harvested, washed twice with 1 × phosphate-buffered saline (PBS), fixed with ice-cold 70% ethanol, and stained with the Tali^®^ Cell Cycle Solution. The cells were analyzed with a Tali^®^ Image-Based Cytometer.

#### Apoptosis Analysis

The apoptosis of HCT116 p53^–/–^ cell transfected with plant miRNA mix (15 μM f.c. for each miRNA) or with the control miRNA was analyzed using Tali^®^ Apoptosis Kit-Annexin V Alexa Fluor^®^ 488 and propidium iodide (Life Technologies). The cells were harvested and resuspended in 1 × Annexin binding buffer, 5 μl of Annexin V Alexa Fluor^®^ 488, and 1 μl of Tali^®^ propidium iodide were addended to the cells. The cells were analyzed with a Tali^®^ Image-Based Cytometer.

#### RNA Extraction and Transcriptomic Analysis

Total RNAs (mRNA + miRNA fraction) were extracted, after the transfection with plant miRNA mix or with the control miRNA, from LAN-1, T98G, HepG2, HCT116 p53^–/–^, and HCT116 p53^+/+^ cell lines by using the RNeasy Plus Mini Kit (Qiagen) and then quantified on a Nanodrop2000 spectrophotometer (Thermo-Scientific). RNA quality was assessed running aliquots on the 2100 Bioanalyzer (Agilent Technologies). Finally, the RNA extracted after 72 h, from HCT116 p53^–/–^ and HCT116 p53^+/+^ cell lines, has been used to prepare the sequencing libraries. The libraries were prepared by using the TruSeq Stranded RNA kit and TruSeq smallRNA kit according to the manufacturer’s instructions (Illumina) and sequenced on a MiSeq Illumina platform.

#### Statistical Analysis of RNA-Seq Data

RNA-Seq reads were processed with a standard bioinformatic analysis pipeline. To ensure the absence of significant sequencing errors or other technical issues, the quality of reads was assessed with the FastQC tool (Andrews, 2010). Reads were aligned to the Ensembl Human Transcripts database (Hubbard et al., 2002) using STAR (Dobin et al., 2013). Read counts were computed both at gene and isoform level. Reads mapping to more than one reference sequence (e.g., multireads) were evaluated and processed with MultiDEA (Consiglio et al., 2016) and RSEM (Li and Dewey, 2011) to control the effects of ambiguous mappings on the significance of statistical results. Normalization was carried out using the method implemented in DESeq2: a scaling factor is computed for each sample as the median of each gene’s scaling ratio over its geometric mean across all samples (Love et al., 2014).

Statistics for differential gene expression profiling between samples treated with plant miRNAs and corresponding controls were carried out by Fisher’s exact test (Upton, 1992) and *p* values adjusted for the false discovery rate (FDR) by Benjamini–Hochberg procedure (Benjamini and Hochberg, 1995). Fold change (FC) was computed as the ratio of normalized read counts. Differential expression events were filtered by adjusted *p* value ≤ 0.05 and fold change ≥ 1.5 (absolute value).

#### RT-qPCR Analysis

Ten nanograms of total RNA was retrotranscribed using TaqMan MicroRNA RT Kit (Life Technologies). Transfected plant miRNA expression levels were measured by TaqMan MicroRNA Assay (Life Technologies) in triplicate, using the ABI PRISM 7900HT platform (Applied Biosystems^®^, Life Technologies^TM^). snU6 expression was used for RNA normalization (code 001973 Life Technologies). Five hundred nanograms of total RNA were reverse transcribed by using QuantiTect^®^ Reverse Transcription kit (Qiagen) to measure target genes expression. RT-qPCR experiments were carried out on HCT116 p53^+/+^ and HCT116 p53^–/–^ cell lines to gage target genes expression by using TaqMan^®^ assays (Life Technologies). The expression levels of target genes were normalized by using the mean expression levels of glyceraldehyde 3-phosphate dehydrogenase (GAPDH) housekeeping gene, selected as the most stable housekeeping gene (between ACTB, GAPDH and RPL13) by the geNorm VBA applet for Microsoft Excel. Negative controls were included without template (NTCs) for each TaqMan assay.

The average of at least three independent experiments was performed and represented in graphs with standard deviation. Student’s *t* test was used for statistical analysis, and *p* < 0.05 was considered to be statistically significant.

#### Luciferase Assay

To demonstrate that the plant miRNAs regulate the expression of MALAT1 and NEAT1, we performed luciferase assays in the HCT116 p53^–/–^ colon carcinoma cell lines. The wild type and mutated fragments containing the region complementary to the 5′ end of the plant miRNA of interest were amplified and cloned into an expression vector for the pMIR luciferase gene [pMIR luciferase reporter vector (Life Technologies)]. The resulting clones were sequenced to verify proper sequence identity.

HCT116 p53^–/–^ cells (2 × 10^5^) were plated in six-well plates and transfected 24 h later (70–80% confluency) with 30 pmol of mtr-miR-5754 [or negative control miRNA (Ambion) or unspecific plant miRNA-ath-miR5014a-3p] together with 1 μg of pMIR-MALAT1 3′UTR and 10 ng of pRL SV40 Renilla luciferase vector (transfection control), or with 30 pmol of gma-miR-4995 [or negative control miRNA (Ambion) or unspecific plant miRNA-ath-miR5014a-3p] together with 1 μg of pMIR-NEAT1 3′UTR and 10 ng of pRL SV40 Renilla luciferase vector (transfection control), using polyethylenimine (PEI, Sigma Aldrich). 48 h later, cells were lysed, and both firefly and Renilla luciferase activities were measured using the Dual-Luciferase Assay System (Promega) and quantified on a TD-20/20 Luminometer (Turner Designs). Firefly luciferase was normalized to Renilla luciferase activity, presented as relative luciferase activity. At least three independent experiments were performed and reported in the graph as the average with the standard deviation. Statistical analysis was performed by using Student’s *t* test. *p* < 0.05 was considered to be statistically significant.

## Results

### Bioinformatic Analysis

A preliminary study was carried out to investigate if plant miRNAs, transfected in human cell lines, can bind gene transcripts using a short 5′ end sequence to target endogenous miRNA recognition motifs. To this aim, we performed a global and a local alignment analysis searching for sequence similarity between human and plant miRNAs. The global pairwise alignment analysis did not show any complete perfect match. However, similarity values obtained were much higher than in the alignment of human miRNAs with a dataset of the random sequences. Only five human miRNAs showed a similarity in the range of 80–90% with plant miRNAs. The 25% (e.g., 661) of human miRNAs showed a similarity to plant in the range of 70–80%, whereas the remaining 75% (e.g., 1920) showed a sequence similarity in the range of 60–70%.

In accordance with the hypothesis that plant miRNAs can have a regulatory effect on mammal genes, we have searched for perfect local matches of plant miRNAs 5′ sequences in the human miRNAs seed (positions 2–8, 2–7, and 3–8), resulting in 3,172 plant miRNAs having a perfect match with 1,606 human miRNAs out of 2,588 analyzed.

These 1,606 miRNAs were used to search for validated gene targets in miRTarBase. The result was a list of 2,203 human genes (Supplementary Table 1—sheet “Plant–Human miRNAs”). This gene list was analyzed using DAVID for statistical enrichment in colon cancer, cell cycle, and cell proliferation. Among the results of these analyses, we identified 20 human miRNAs because of their role in cell cycle regulation as an oncogene, tumor suppressors, biomarkers for prognosis or diagnosis of colon cancer (Piepoli et al., 2012; Zhang K. et al., 2018) as it is shown in Supplementary Figure 1. Using our data warehouse, we searched for plant miRNAs with a sequence at their 5′ end (positions 2–8, 2–7, and 3–8) perfectly matching the seed sequence of these 20 human miRNAs (Table 1) obtaining seven plant miRNA, namely, gma-miR-160, gma-miR-4995, gma-miR-4368, gma-miR-5677, gma-miR-4351, zma-miR-172, and mtr-miR-5754, which were then used for cell transfection experiments (Figure 1).

### Reduction in Cell Proliferation Induced by Plant miRNAs

To test if the selected plant miRNAs (Table 1) could be able to affect human cell proliferation rate, we performed CCK-8 assay upon plant miRNA transfection. We used different human cancer cell lines considering the p53 tumor suppressor gene status because it is a key player in cell cycle control and apoptosis and p53 mutation is the most common genetic abnormality found in human cancer. Moreover, mutant p53 acquires an oncogenic gain-of-function (GOF) activity that promotes the transformation of cancer cells. Cell lines used were two neuronal cancer cell lines (neuroblastoma LAN-1-p53^–/–^ and glioblastoma T98G with mutant p53), two cancer intestinal cell lines (colorectal carcinoma HCT116 cells with p53^+/+^ and colorectal carcinoma HCT116 cells-p53^–/–^) and the human liver cancer cell line HepG2 with p53^+/+^. Cells were transfected with control miRNA or with a mix of the seven exogenous selected plant miRNAs (plant miRNA mix) for 24, 48, and 72 h, and their ectopic expression was monitored by RT-qPCR (Supplementary Figure 2).

**FIGURE 2:**
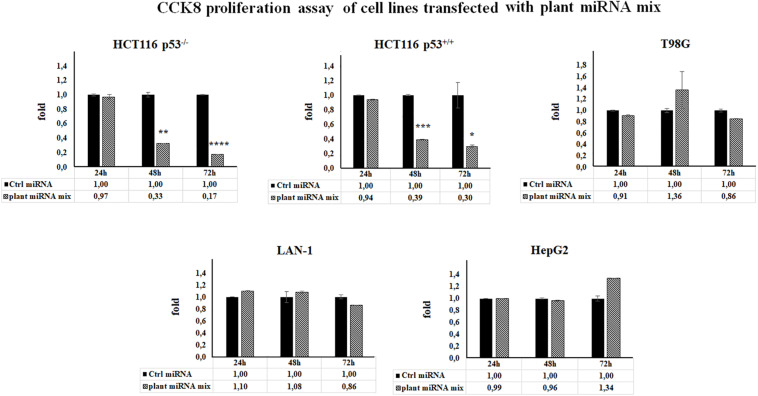
Plant microRNAs (miRNAs) are able to modulate human cell proliferation rate. Cell proliferation was measured by Cell Counting Kit-8 (CCK-8) assay in cell lines transfected for 24, 48, and 72 h with control miRNA or with a mix of the seven selected plant miRNAs (15 μM f.c. for each; gma-miR-160, gma-miR-4995, gma-miR-4368, gma-miR-5677, gma-miR-4351, zma-miR-172, and mtr-miR-5754). Data presented show the average of three independent experiments with standard deviation (*p < 0.05; **p < 0.005; ***p < 0.0005, and ****p < 0.00005).

The results of CCK-8 proliferation assay showed that the plant miRNA mix induced a cell proliferation reduction in HCT116 p53^+/+^ and HCT116 p53^–/–^ cell lines ranging from 70 to 80%, independently of p53 status (Figure 2).

**FIGURE 3:**
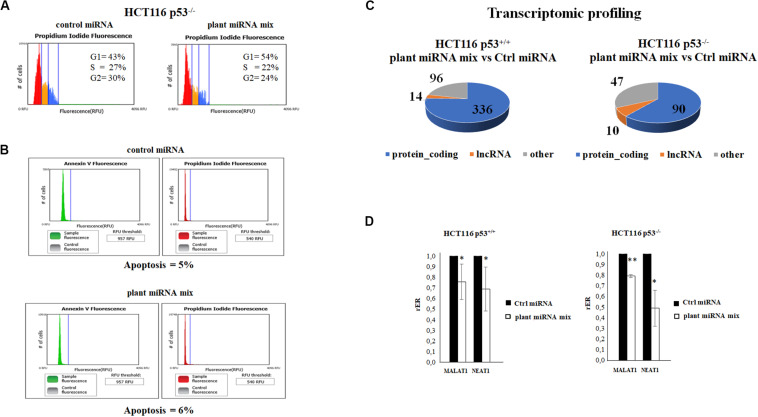
Plant microRNAs (miRNAs) induce the arrest of the cells in G1 and modulate the transcriptomic profile. **(A)** Cell cycle analysis of HCT116p53^–/–^ transfected for 48 h with control miRNA or with a mix of the seven selected plant miRNAs (15 μM f.c. for each; gma-miR-160, gma-miR-4995, gma-miR-4368, gma-miR-5677, gma-miR-4351, zma-miR-172, and mtr-miR-5754). **(B)** The apoptosis of HCT116 p53^–/–^ cell transfected with plant miRNA mix (15 μM f.c. for each plant miRNA) or with the control miRNA was analyzed using Tali^®^ Apoptosis Kit–Annexin V Alexa Fluor^®^ 488 and propidium iodide. **(C)** The pie charts show the proportions of transcript types for statistically significant differentially expressed genes in HCT116 p53^+/+^ and HCT116 p53^–/–^ from RNA-Seq data analysis: protein coding, long non-coding RNAs (lncRNAs), others. **(D)** Reverse transcription quantitative PCR (RT-qPCR) of metastasis-associated lung adenocarcinoma transcript 1 (MALAT1) and nuclear paraspeckle assembly transcript 1 (NEAT1) lncRNAs in HCT116 p53^+/+^ and HCT116 p53^–/–^ cell lines transfected for 72 h with control miRNA or with a mix of the seven selected plant miRNAs (gma-miR-160, gma-miR-4995, gma-miR-4368, gma-miR-5677, gma-miR-4351, zma-miR-172, and mtr-miR-5754). Data are shown as the average with a standard deviation of three independent experiments (*p < 0.05 and **p < 0.005).

We examined the cell cycle profile and the apoptosis status of the HCT116p53^–/–^ transfected with the plant miRNA mix by propidium iodide and Annexin V assay followed by cytometry analysis. As shown in Figures 3A,B, plant miRNA mix did not induce apoptosis but an arrest of the cells in G1 and a decrease in the percentage of the cells in S and G2 phases compared to the control cells.

### RNA Sequencing Results of Transfected Cell Lines and Functional Enrichment Analysis of Differentially Expressed Transcripts

To investigate plant miRNA mix effects in transcriptome profile, we selected the HCT116 p53^+/+^ and HCT116 p53^–/–^ colon cancer cell lines because, among all, they showed a significant decrease in proliferation. RNA-Seq experiments were carried out after the transfection with the plant miRNA mix. Total RNA was depleted from ribosomal RNA (rRNA), and directional RNA sequencing produced an average of 17.5 million reads per sample, out of which 60% were aligned to the reference genome. The sequencing showed optimal coverage and sequencing depth with low rRNA contamination. The RNA-Seq data analysis identified 23,864 annotated transcripts as present in at least one of the sequenced samples. The Fisher’s exact test identified a total of 446 differentially expressed (DE) gene transcripts (*p* ≤ 0.05 after FDR correction, absolute fold change ≥ 1.5) in the HCT116 p53^+/+^ cell line (252 downregulated and 194 upregulated; Figure 3C and Supplementary Table 3) and 123 (56 downregulated and 67 upregulated) in the HCT116 p53^–/–^ cell line (Figure 3C and Supplementary Table 4). The functional enrichment analysis of downregulated genes in the HCT116 p53^+/+^ cell line returned a significant result in the pathway “extracellular matrix organization” (*p* value, 8.0E-3) of the Reactome Pathway Database (Jassal et al., 2020).

This result is valuable because this pathway performs many functions that influence cell behaviors, such as migration, adhesion, and proliferation, and modulates cell death and differentiation (Hynes, 2009).

Upregulated genes were enriched in more pathways, in particular, in the BIOCARTA pathway, “role of mitochondria in apoptotic signaling” (*p* value, 1.9E-2), and in Kyoto Encyclopedia of Genes and Genomes (KEGG) pathways (Kanehisa and Goto, 2000) such as “p53 signaling” (*p* value, 9.4E-2), “transcriptional misregulation in cancer” (p, 4.6E-4), “chromatin organization” (R-HSA-3214858; *p* value, 1.9E-15), “RMTs methylate histone arginines” (*p* value, 1.2E-15), “HDACs deacetylate histones” (*p* value 1.8E-14), and “condensation of prophase chromosomes” (*p* value, 2.8E-14). As for the HCT116 p53^–/–^ cell line, a positive result was obtained only for upregulated genes that showed significant enrichment in the KEGG pathway “transcriptional misregulation in cancer” (*p* value, 1.5E-2).

We also looked for genes DE in both the cell lines, HCT116 p53^+/+^ and HCT116 p53^–/–^ (Supplementary Tables 3, 4). An intriguing result was the observation of the downregulation of two long non-coding RNA, which are key players in many different types of cancers, MALAT1 and NEAT1.

### Plant miRNAs Suppressed the Expression of the Oncogenic lncRNA MALAT1 and NEAT1 in Colon Cancer Cell Lines

Metastasis-associated lung adenocarcinoma transcript 1 and NEAT1 lncRNAs were significantly downregulated both in HCT116 p53^+/+^ and HCT116 p53^–/–^ transfected with plant miRNA mix compared to controls (adjusted *p* < 2.51e-07). We first validated the MALAT1 and NEAT1 differential expression by RT-qPCR that confirmed the RNA-Seq results in both cell lines (Figure 3D).

We chose these genes for further analyses because they map in adjacent gene loci within human chromosome 11q13.1 (NEAT1 is placed 30 kb 5′ side from MALAT1) and their synergic regulation of gene expression has been suggested (Jen et al., 2017). Moreover, MALAT1 is one of the first identified cancer-associated lncRNAs; it is upregulated in several cancers, and its deregulation is considered a prognostic biomarker for particularly aggressive tumors (Hirata et al., 2015; Ma et al., 2015; Chen et al., 2017; Zhang X. et al., 2017; Li et al., 2018b; Sun and Ma, 2019).

Nuclear paraspeckle assembly transcript 1 modulates DNA-damage response in cancer cells, and its upregulation was strongly correlated with poor prognosis and negative clinical–pathological parameters such as the numbers of tumor nodes, metastasis, tumor–node–metastasis (TNM) stage, and cell migration and invasion in different tumors (Guo et al., 2015; Ghafouri-Fard and Taheri, 2019).

**FIGURE 4:**
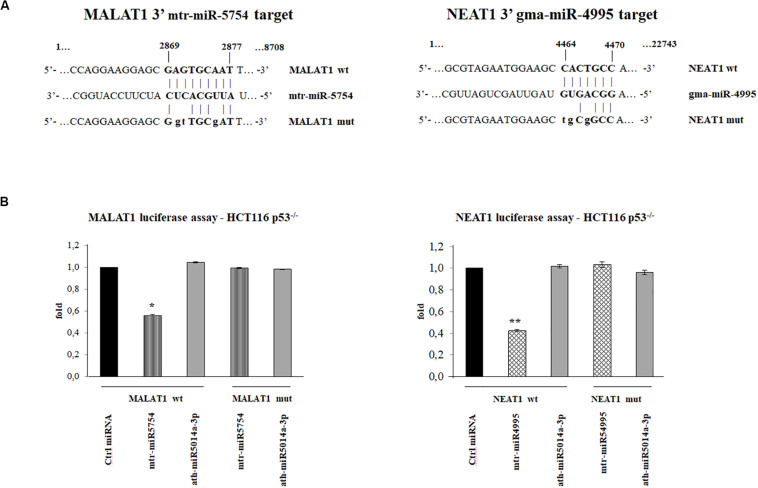
Plant microRNAs (miRNAs) mtr-miR-5754 and gma-miR-4995 regulate metastasis-associated lung adenocarcinoma transcript 1 (MALAT1) and nuclear paraspeckle assembly transcript 1 (NEAT1) stability. **(A)** Schematic representation of the putative wild-type and mutated sequences at 3′-end of MALAT1 and NEAT1, complementary to 5′ end of the plant mtr-miR-5754 and to gma-miRNA-4995, which were cloned into the pMIR luciferase reporter construct containing downstream the luciferase gene. In bold is the sequence alignment between the plant miRNAs 5′ end sequences and their specific binding regions. The letters in lowercase represent the mutagenized nucleotides. **(B)** HCT116 p53^–/–^ cells were transfected with pMIR luciferase reporter construct containing MALAT1 3′ region together with negative control miRNA, mtr-miR-5754, or unspecific plant miRNA. The HCT116 p53^–/–^ cells were transfected with pMIR luciferase reporter construct containing NEAT1 3′ region together with negative control miRNA, gma-miR-4995, or unspecific plant miRNA. 48 h after transfections, cells were lysed, and luciferase activity was determined. Transfection efficacy was normalized by Renilla luciferase activity. Data represent the averages of three independent experiments with their standard deviations (*p < 0.05 and **p < 0.005).

To investigate which plant miRNA used for transfections might have a sequence-specific target on MALAT1 and NEAT1, we used miRanda. Putative target sites were identified using as query sequences the seven plant miRNAs selected. In particular, we identified a putative target in MALAT1 (Vega transcript ID OTTHUMT00000389143) for mtr-miR5754 in position 2869, with minimum free energy (MFE) of -27.15 kcal/mol and a putative target in the NEAT1 transcript (Vega transcript ID OTTHUMT00000389142) for gma-miR4995 in position 4464 with MFE of -21 kcal/mol, as shown in Figure 4A.

### Mtr-miR-5754 and gma-miR-4995 Directly Target lncRNA MALAT1 and NEAT1

To demonstrate the direct effect of mtr-miR-5754 and gma-miR-4995 on the expression level of MALAT1 and NEAT1, we performed luciferase reporter assay. We cloned the sequence containing the putative target sequences of mtr-miR-5754 and gma-miR-4995 identified by miRanda, downstream of the *luc2* luciferase gene, under the control of the human constitutive promoter (pMIR-MALAT1 or pMIR-NEAT1), and transfected them in HCT116 p53^–/–^ cell line with negative control miRNA (Ambion), mtr-miR-5754, or gma-miR-4995. The effectiveness of the transfections was confirmed by RT-qPCR (data not shown). The luciferase reporter assays showed that both mtr-miR-5754 and gma-miR-4995 significantly downregulate the firefly luciferase activity of pMIR-MALAT1 and pMIR-NEAT1, respectively (about 50%), suggesting a direct role of mtr-miR5754 and gma-miR4995 in regulating MALAT1 and NEAT1 RNA levels (Figure 4B). To confirm the specificity of the interaction between the plant mtr-miR-5754 and gma-miR-4995 and their respective targets in MALAT1 and NEAT1, these latter sequences were mutagenized, and in this form, mtr-miR-5754 and gma-miR-4995 failed to suppress the firefly luciferase activity (Figures 4A,B). Moreover, in the luciferase assay, we used also another unspecific plant miRNA, and this latter failed to suppress the firefly luciferase activity (Figures 4A,B).

**FIGURE 5:**
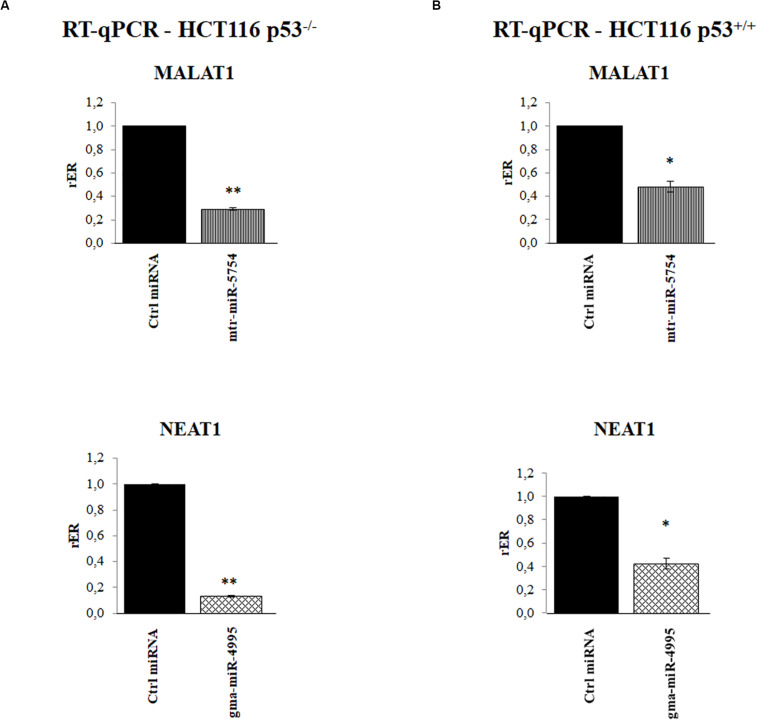
Plant microRNAs (miRNAs) mtr-miR-5754 and gma-miR-4995 regulate metastasis-associated lung adenocarcinoma transcript 1 (MALAT1) and nuclear paraspeckle assembly transcript 1 (NEAT1) stability. **(A,B)** Reverse transcription quantitative PCR (RT-qPCR) of MALAT1 and NEAT1 long non-coding RNAs (lncRNAs) in HCT116 p53^–/–^ and HCT116 p53^+/+^ cell lines transfected with control miRNA, with mtr-miR-5754 or with gma-miR-4995 for 48 h. Data are shown as the average with a standard deviation of three independent experiments (*p < 0.05; **p < 0.01).

**FIGURE 6:**
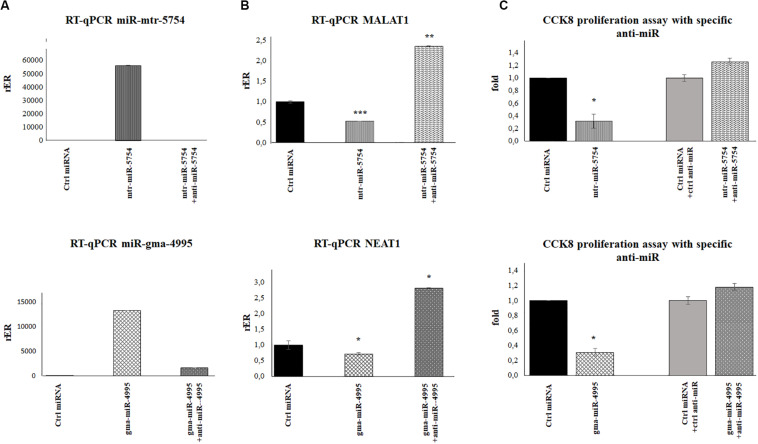
Plant mtr-miR-5754 and miRgma-miR-4995 inhibitors reversed the effect of mtr-miR-5754 and miRgma-miR-4995 on metastasis-associated lung adenocarcinoma transcript 1 (MALAT1) and nuclear paraspeckle assembly transcript 1 (NEAT1) expression. **(A)** HCT116p53^–/–^ cells were transfected with control microRNA (miRNA), mtr-miR-5754, or gma-miR-4995 and after 24 h with anti-miR5754 or anti-miR4995, respectively, which suppressed the plant miRNA levels in the cells. **(B)** Reverse transcription quantitative PCR (RT-qPCR) of MALAT1 and NEAT1 in HCT116p53^–/–^ cells transfected with control miRNA, mtr-miR-5754, or gma-miR-4995 and after 24 h with anti-miR5754 or anti-miR4995, respectively (*p < 0.05; **p < 0.005; and ***p < 0.0005). **(C)** Cell Counting Kit-8 (CCK-8) assay of HCT116p53^–/–^ cells transfected with control miRNA, mtr-miR-5754, or gma-miR-4995 and after 24 h with anti-miR5754 or anti-miR4995, respectively.

To further validate our results, we explored the effect of mtr-miR-5754 or gma-miR-4995 overexpression by their transient transfection in HCT116 p53^–/–^ and HCT116 p53^+/+^ cell lines. RT-qPCR showed that the overexpression of mtr-miR-5754 and gma-miR-4995 reduced the steady-state level of MALAT1 and NEAT1 RNA level in both cell lines (Figures 5A,B). Coherently, the overexpression of mtr-miR-5754 and gma-miR-4995 decreased the cell proliferation of colon carcinoma HCT116 cell lines (Figure 6C).

Altogether, our results demonstrate that plant miRNAs can alter the stability of the oncogenic lncRNA MALAT1 and NEAT1.

To further demonstrate that mtr-miR5754 and gma-miR4995 directly downregulate MALAT1 and NEAT1 expression, we transfected HCT116p53^–/–^ cells before with miR5754 or miR4995 and after 24 h with anti-miR5754 or anti-miR4995, respectively. Anti-miR5754 or anti-miR4995 efficiently silenced their respective miRNA (Figure 6A), and simultaneously, the expression level of MALAT1 and NEAT1 significantly increased (Figure 6B). Coherently, the proliferation rate of HCT116p53^–/–^cells transfected with miR5754 or miR4995 with their respective anti-miR increased, as demonstrated by CCK-8 assay (Figure 6C).

**FIGURE 7:**
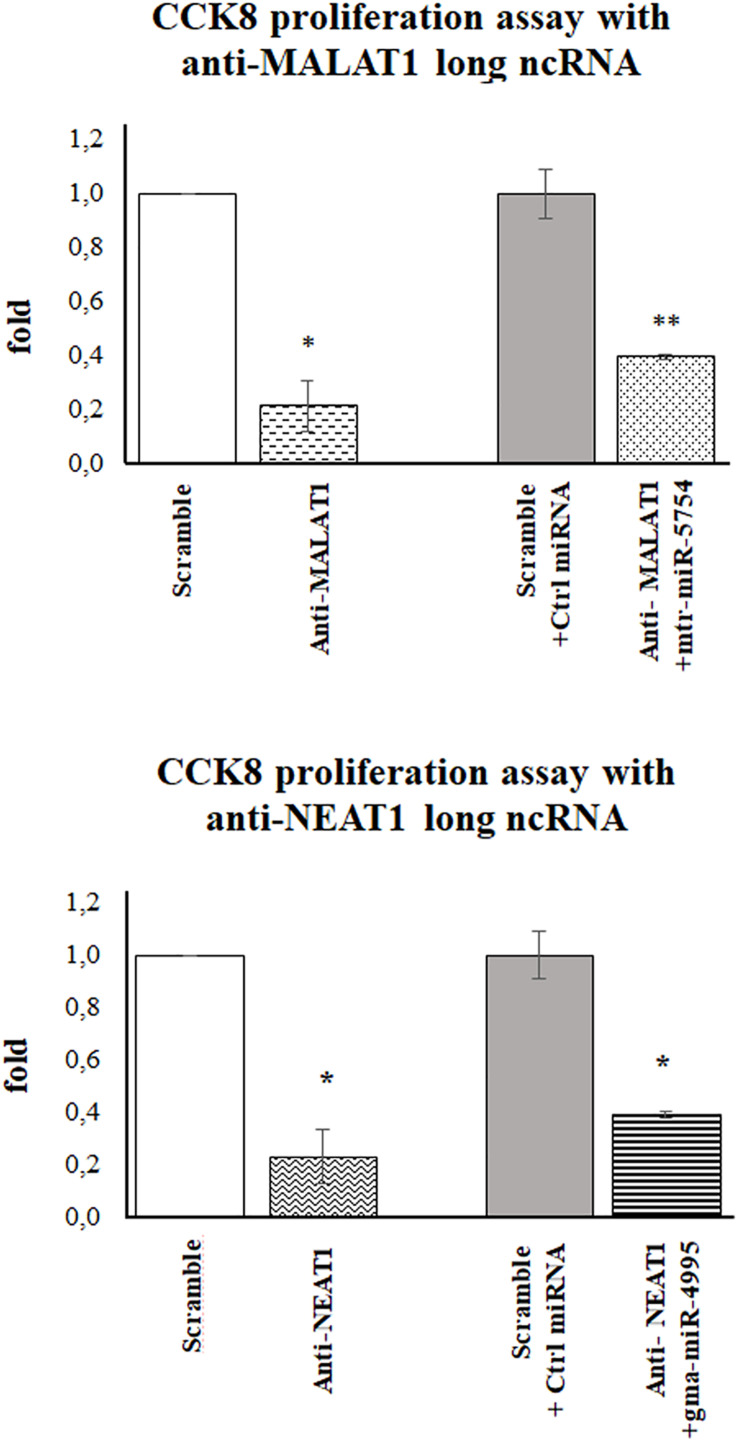
Plant mtr-miR-5754 or gma-miR-4995 reduced cell proliferation through metastasis-associated lung adenocarcinoma transcript 1 (MALAT1) and nuclear paraspeckle assembly transcript 1 (NEAT1) downregulation. Cell Counting Kit-8 (CCK-8) assay of HCT116p53^–/–^ cells transfected with scramble small interfering RNA (siRNA), MALAT1- or NEAT1-specific siRNA, and after 24 h with plant mtr-miR5754 or gma-miR4995 (*p < 0.05 and **p < 0.005).

To evaluate if mtr-miR5754 or gma-miR4995 caused the arrest of HCT116 p53^–/–^ cell proliferation through MALAT1 and NEAT1 downregulation, their expression levels were first silenced by using specific siRNA and then transfected with plant mtr-miR5754 or gma-miR4995. RT-qPCR demonstrated that MALAT1 and NEAT1 were successfully silenced by their specific siRNA and that mtr-miR5754 and gma-miR4995 were efficiently transfected (Supplementary Figures 3A,B). As expected, MALAT1 or NEAT1 silencing induced a decrease in cell proliferation (Figure 7), which did not decrease further when we transfected mtr-miR5754 or gma-miR4995, suggesting that these plant miRNAs can reduce cell proliferation by acting mainly on MALAT1 or NEAT1 expression levels.

## Discussion

Several papers elucidate the protective role of vegetables against cancer and related physiological conditions, such as chronic inflammatory diseases and aging. Moreover, epidemiological studies report that most of the chronic diseases plaguing industrialized countries can be drastically reduced with better nutrition and lifestyle changes.

Recently, numerous scientific studies have demonstrated the presence of plant miRNAs in body fluids: serum, saliva, urine, and in gastric and colon mucosa (Minutolo et al., 2018; Li et al., 2019; Link et al., 2019) and demonstrated that these circulating miRNAs might have a significant involvement in miRNA-mediated gene expression regulation. On the other hand, it has long been known that ingested RNA from food sources in nematodes and insects can control their gene expression (Whangbo and Hunter, 2008). Chin et al. (2016) demonstrated that plant miR159 can be identified in human sera and can affect cancer far from the gastrointestinal tract, such as breast cancer, and that its level is inversely associated with breast cancer progression and incidence. Moreover, they reported that the oral administration of this plant miRNA significantly suppresses the growth of xenograft breast tumors in mice. More interestingly, they report that miR159 is predominantly detected in the extracellular vesicles (EVs) in human sera. Accumulating evidences demonstrated that plant miRNAs could be secreted via EVs, such as exosomes, transferred inside host cells, and modulated cell function, supporting the idea that extracellular miRNAs are considered effectors and messengers in intercellular communication (Teng et al., 2018). Indeed, it was demonstrated that exosome-like nanoparticles (EPDELNs) of edible plant could determine EPDELN-mediated interspecies communication by eliciting anti-inflammatory cytokines genes, anti-oxidation, and activation of Wnt signaling, which are critical for preserving intestinal homeostasis (Mu et al., 2014). In exosome-like nut nanovesicles, plant miR159a and miR156c reduce the level of tumor necrosis factor (TNF) receptor superfamily member 1a (Tnfrsf1a) transcript leading to a decrease in TNF-α signaling pathway and inflammatory markers (Aquilano et al., 2019). Moreover, it was recently reported that MIR167e-5p represses intestinal cell proliferation by targeting β-catenin (Li et al., 2019). More recently, Minutolo et al. demonstrated that the natural oeu-sRNAs decrease the protein expression of hsa-miR34a mRNA targets, reducing proliferation and increasing apoptosis in different tumor cells. These miRNAs were extracted from *Olea europaea* drupes and had been previously reported to have functional homology to hsa-miR34a (Minutolo et al., 2018).

The novelty introduced by the results of our study is that, for the first time, we provide evidence that not only protein-coding transcripts but also lncRNAs can be targeted and inhibited in their function by plant miRNAs. These new findings further support the intriguing possibility of a “horizontal” cross-kingdom interaction in which plant miRNAs may influence human gene expression mimicking endogenous miRNAs through a sequence-specific targeting. Mechanistically, there are many fundamental similarities between plant and animal miRNAs, and although we did not find evidence (data not shown) for miRNA plant sequences perfectly identical to human miRNAs, our results demonstrated that several plant miRNAs share with human miRNAs a perfect identity in the seed region, which is the essential part for human target recognition (Bartel, 2009). The lncRNAs are increasingly emerging as crucial regulators of many physiological processes, and their malfunction might have a negative impact on different pathologies, including cancer. LncRNAs are both positive and negative regulators of protein-coding genes, through lncRNA:RNA, lncRNA:protein, and lncRNA:chromatin interactions. They are precursors of sRNAs and play pivotal roles in several molecular mechanisms including RNA processing, epigenetic modulation, transcriptional and posttranscriptional regulation, nuclear organization, and nuclear-cytoplasmic trafficking.

In this paper, we selected *in silico* seven plant miRNAs (gma-miR160, gma-miR4995, gma-miR4368, gma-miR5677, gma-miR4351, zma-miR172, and mtr-miR5754) showing an identical sequence at their 5′ end with the seeds of 20 human miRNAs that target genes involved in cell cycle regulation, in tumor progression, and in formation of metastases. Coherently, we observed that the mix of these plant miRNAs, transfected in different cell lines, induced a p53-independent decrease in cell proliferation with a considerable impact on the expression of many genes in cancer intestinal HCT116 cells.

Noteworthy in this study is that we demonstrated that mtr-miR5754 and gma-miR4995 directly target the transcripts of two lncRNAs, MALAT1, and NEAT1, which are both associated with almost all type of tumors and in particular with the metastatic tumor process. It is well known that pathological processes are triggered by abnormal MALAT1 expression level due to endogenous or exogenous inducers, and several studies in humans have shown that lncRNA can act as miRNA sponge, thus preventing the inhibition of translation of target mRNAs (López-Urrutia et al., 2019).

Our results about the significant enrichment of upregulated genes in the HCT116 cell line support a specific influence of transfected plant miRNAs on all those processes related to chromatin and epigenetic modifications, many of which were mediated by lncRNAs. Moreover, the importance of the role and deregulation of ncRNAs in cancer is well documented, and indeed for this reason, they are also referred to as potential targets for new cancer therapies (Chen et al., 2017).

Metastasis-associated lung adenocarcinoma transcript 1 was one of the first identified cancer-associated lncRNA in the lung, bladder, breast, cervical, colon, colorectal, endometrial, esophageal, gastric, lymphoblastoid, ovarian, and prostate cancer. It was also associated with hepatocellular carcinoma, melanoma, multiple myeloma, neuroblastoma, glioblastoma, osteosarcoma, pituitary adenoma, and renal cell carcinoma (Hirata et al., 2015). Moreover, cumulative evidence demonstrates that MALAT1 plays pivotal roles in coordinating several pathophysiological processes. In cardiovascular disease (myocardial infarction, diabetes-mellitus-related vascular complications), it is overexpressed, and dysregulation of its expression has also been demonstrated in neurological disorders (e.g., stroke, Alzheimer’s and Parkinson’s disease, and retinal neurodegeneration) (Zhang X. et al., 2017).

In physiological condition, MALAT1 has multiple molecular functions. It is known that it is retained in the nucleus, where it forms molecular scaffolds for ribonucleoprotein complexes. Therefore, it has been supposed that MALAT1 might modulate the expression of several genes, as those involved in cell cycle regulation, where it is crucial for G1/S and mitotic progression (Tripathi et al., 2013).

In concert with MALAT1, also NEAT1 is emerging as a novel biomarker and crucial regulator in the pathogenesis of several human tumors although with distinct roles in solid tumors versus hematological malignancies (Ghafouri-Fard and Taheri, 2019). Similarly to MALAT1, NEAT1 is localized in the nucleus where it constitutes the core structural compound of paraspeckle suborganelles. In several genomic loci, NEAT1 colocalize with MALAT1 with independent but correlative activities, and it is involved in the regulation of different genes, including some genes controlling cancer progression.

Findings related to protein-coding genes might represent a useful resource for future deeper investigations. The total number of DE genes are indeed 446 in HTC116p53^+/+^ cells and 123 in HTC166-p53^–/–^. Our analysis with DAVID showed that they might have important roles in many different processes and pathways, providing for the first time pieces of evidence that plant miRNAs can have a broad impact on colon cancer gene expression.

In conclusion, the proposed approach considered as a pre-requirement the identity of sequences between plant and human miRNAs in canonical seed positions. Based on this assumption, we provide the first evidence that plant mtr-miR5754 and gma-miR4995 can significantly reduce the level of MALAT1 and NEAT1 in colon cancer cell lines. The mechanism is sequence specific and has an effect similar to that of endogenous miRNAs. Their action on intestinal cancer cell proliferation is p53 independent. In addition, the expression of protein-coding genes might be influenced by the same mechanism. The bioinformatic functional analysis of DE genes supports the hypothesis that a broad range of biological processes could be targeted by plant miRNAs such as apoptosis, cell cycle progression, metabolic processes, response to organic substances and hormone stimulus, etc. (Supplementary Table 2). These findings can offer opportunities for further development and biotechnological applications as, for example, in nutritional research and in supporting antineoplastic strategies.

## Data Availability Statement

The RNA-seq data sets (4 in total) have been submitted to SRA – BioProject (PRJNA629374).

## Author Contributions

FM and MC performed the biological experiments. DC conceived the idea, contributed to the database model design, developed the ETL procedures, and performed the bioinformatic analysis. FM, MC, ES, and AT planned the experiments. AC, DD’E, FL, and SL contributed to the database model design, developed the data warehouse, and performed the bioinformatic analysis of NGS data, statistical analysis of results, and production of plots. All the authors interpreted the data, drafted, and edited the manuscript. All the authors critically revised the manuscript for intellectual content.

## Conflict of Interest

The authors declare that the research was conducted in the absence of any commercial or financial relationships that could be construed as a potential conflict of interest.

## Footnotes

1 http://ibbr.cnr.it/mgd/

2 https://plants.sc.egov.usda.gov/java/index.jsp

## Abbreviations

gma: glicine max
zma: *Zea mays*
mtr: *Medicago truncatula.*
