# Supplementary material for: Plant miRNAs Reduce Cancer Cell Proliferation by Targeting MALAT1 and NEAT1: A Beneficial Cross-Kingdom Interaction
Source: Front Genet. 2020 Sep 18;11:552490. doi: 10.3389/fgene.2020.552490 (PMC7531330; doi:10.3389/fgene.2020.552490)
Supplement: Supplementary file 1 [file Data_Sheet_1.docx]

**LEGENDS TO SUPPLEMENTARY FIGURES**

**Supplementary Figure 1. Statistical enrichment analysis in KEGG.** Genes analysed were the validated targets, as reported in miRTarBase, of twenty selected human miRNAs with the seed regions identical to the plant miRNAs used for the transfection experiments (e.g., gma-miR160, gma-miR4995, gma-miR4368, gma-miR5677, gma-miR4351, zma-miR172, mtr-miR5754). The analysis was carried using DAVID v6.8.

**Supplementary Figure 2. The transfection of each plant miRNA in LAN1, T98G, HepG2, HCT116-p53^+/+^** **and HCT116-p53^-/-^ was checked by RT-qPCR experiments.** RT-qPCR of gma-miR-160, gma-miR-4995, gma-miR-4368, gma-miR-5677, gma-miR-4351, zma-miR-172, mtr-miR-5754 transfected for 48 hours in LAN-1-p53-/-, T98G, HepG2, HCT116 p53^+/+^ and HCT116p53^/-^ cell lines. Data are shown as the average with a standard deviation of three independent experiments.

**Supplementary Figure 3. The transfection of siRNA and plant miRNA. (A)** RTqPCR of MALAT1 and NEAT1 in HCT116p53^-/-^ cells transfected with scramble siRNA, MALAT1 or NEAT1 specific siRNA and after 24h with plant mtr-miR5754 or gma-miR4995. **(B)** RTqPCR of plant mtr-miR5754 or gma-miR4995 in HCT116p53^-/-^ cells transfected with scramble siRNA plus control miRNA, MALAT1 or NEAT1 specific siRNA and after 24h with plant mtr-miR5754 or gma-miR4995 (** p-value < 0.005; *** p-value < 0.0005, **** p-value < 0.00005).

.
