# Supplementary figures and images for: Plant miRNAs Reduce Cancer Cell Proliferation by Targeting MALAT1 and NEAT1: A Beneficial Cross-Kingdom Interaction
Source: Front Genet. 2020 Sep 18;11:552490. doi: 10.3389/fgene.2020.552490 (PMC7531330; doi:10.3389/fgene.2020.552490)

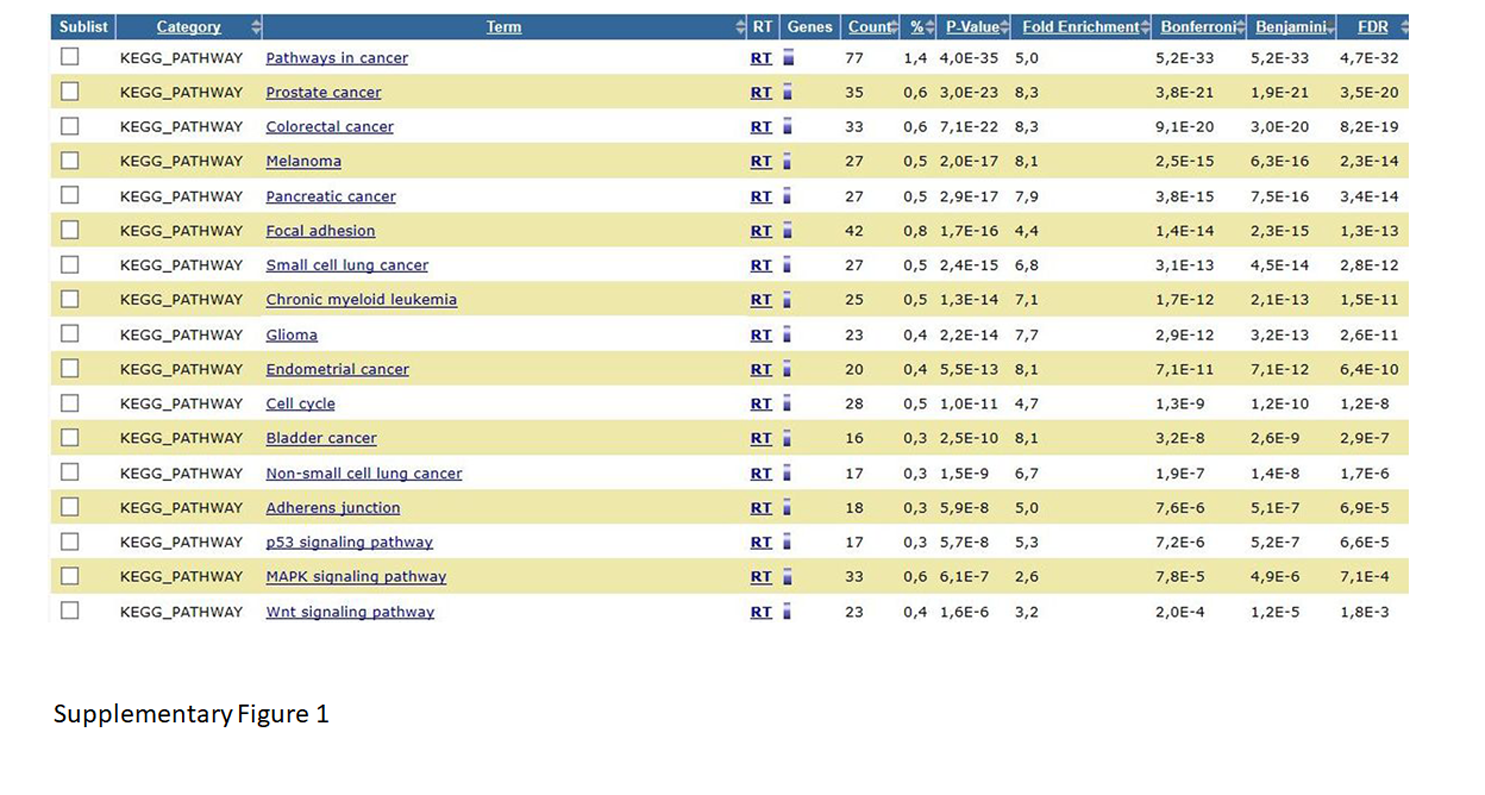

Supplement: Supplementary file 2 [file Image_1.TIF]

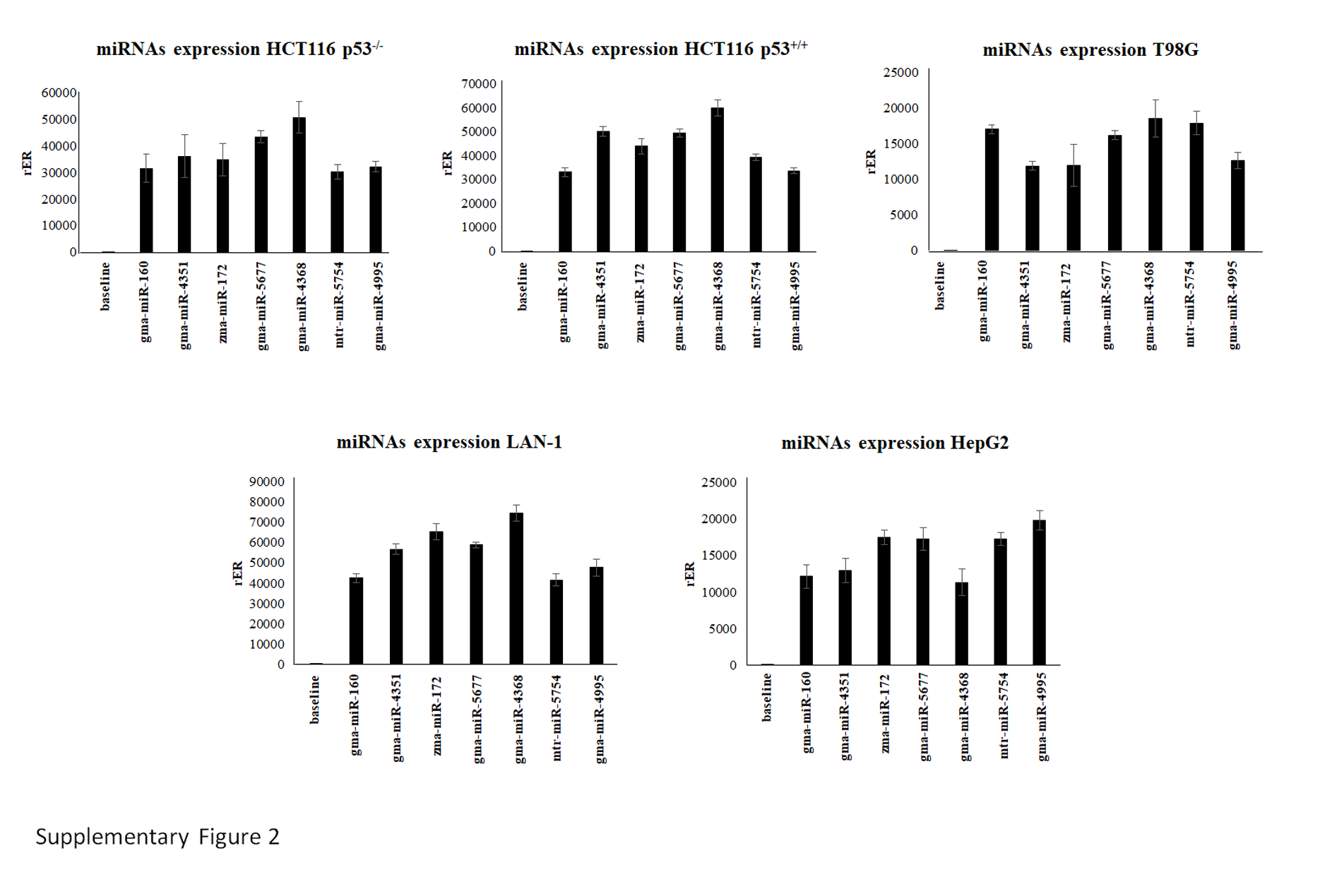

Supplement: Supplementary file 3 [file Image_2.TIF]

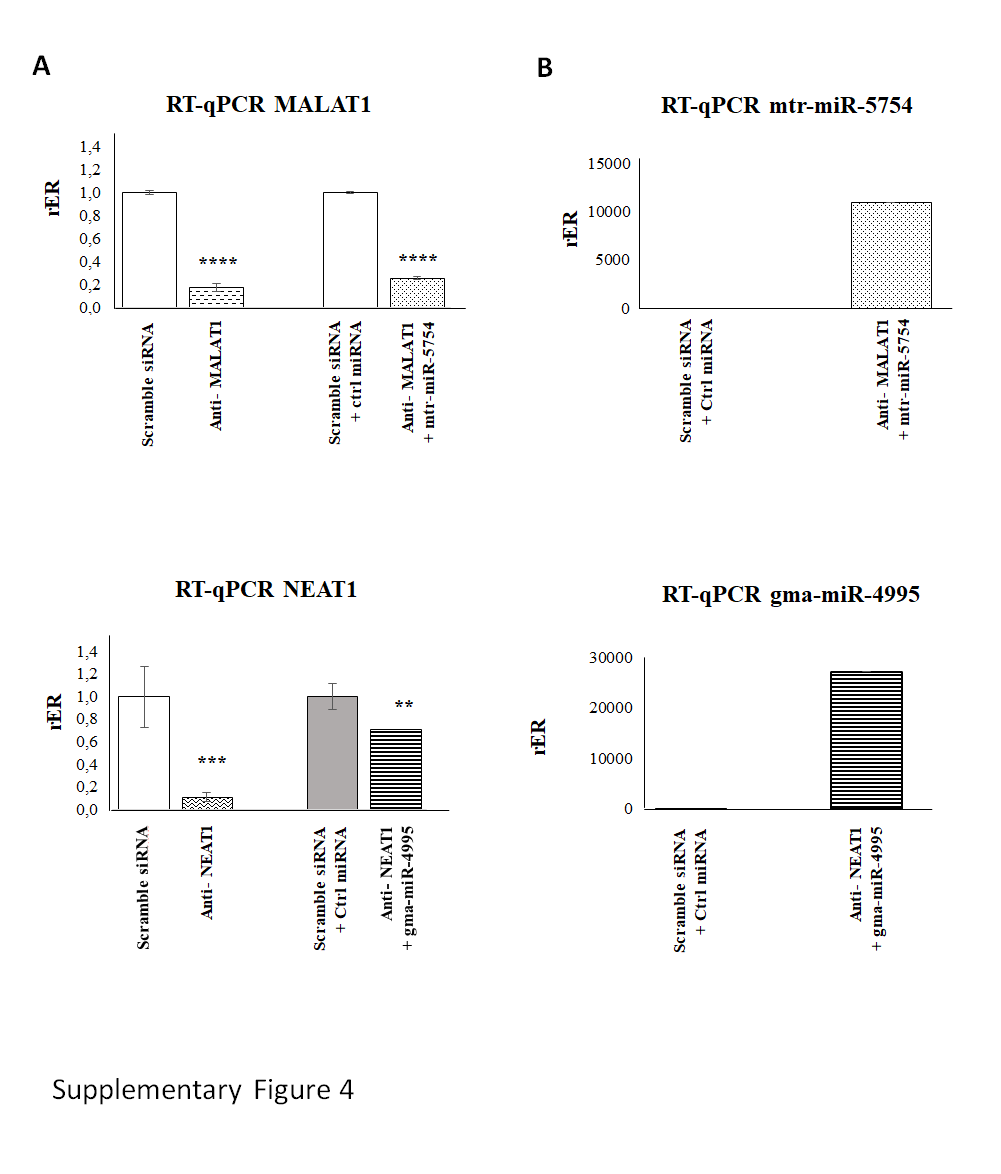

Supplement: Supplementary file 4 [file Image_3.TIF]
